# From policy to person-centred care: insights into the development of complications of excess weight clinics in England

**DOI:** 10.1186/s12913-026-14689-7

**Published:** 2026-05-13

**Authors:** Elysa Ioannou, Karen Coulman, James Nobles, Megan Garside, Lucie Nield, Paige Davies, Karina Kinsella, Louisa Ells, Catherine Homer

**Affiliations:** 1 School of Sport and Physical Activity, Sheffield Hallam University, Sheffield, UK; 2 Population Health Sciences, Bristol Medical School, University of Bristol, Bristol, UK; 3 School of Health, Leeds Beckett University, Leeds, UK; 4 Obesity Institute, Leeds Beckett University, Leeds, UK; 5 School of Health and Social Care, Sheffield Hallam University, Sheffield, UK; 6 School of Medicine and Population Health, University of Sheffield, Sheffield, UK

**Keywords:** Childhood, Children and young people, Obesity, Severe obesity, Weight management, Complications of excess weight, Policy development, Healthcare, Patient centred care

## Abstract

**Background:**

The Complications of Excess Weight (CEW) Programme, funded centrally by NHS England, aims to provide innovative, person-centred, holistic care for children and young people living with severe obesity and associated complications (e.g. hypertension, type 2 diabetes, depression). Twenty-one CEW services were established across England in 2021, rising to 38 in 2024. This qualitative study aimed to gather the perspectives of senior professionals involved in the design, development, and commissioning of the CEW programme.

**Methods:**

Eleven online semi-structured interviews were completed between April and November 2024. Participants included senior officials from NHS England, the Department for Health and Social Care, the Office for Health Improvement and Disparities, specialist weight management clinicians, and a national children’s charity. Interview questions examined stakeholder involvement, the policy backdrop, the intended purpose and expectations of the CEW programme, evaluation expectancies, and future directions. Data were analysed thematically.

**Results:**

Participants described how the CEW programme was developed to meet the needs of a vulnerable population while developing a robust evidence base, aligning with aspirations of the NHS Long Term Plan. The CEW service specification was designed at a high-level to allow local flexibility in implementation and delivery approaches. Key concepts included a biopsychosocial multi-disciplinary approach to manage obesity and complications. Despite systemic challenges, the dedication of staff and involvement of patients supported CEW delivery.

**Conclusions:**

This study offers insight into the development of CEW services from a range of key stakeholders. The importance of public involvement to deliver a complex service within the context of perceived challenges of a fragmented and underfunded system are highlighted. There is a need for sustained investment in CEW, which incorporates a holistic multidisciplinary design to ensure equitable and effective healthcare provision for this vulnerable patient group.

**Supplementary Information:**

The online version contains supplementary material available at 10.1186/s12913-026-14689-7.

## Background

In England, 9.4% of children aged 4–5 years and 22.1% children aged 10–11 years are living with obesity (BMI centile ≥ 95), and 2.6% of 4–5-year-olds and 5.5% of 10–11-year-olds are living with severe obesity (BMI centile ≥ 99.6) [1]. This has increased from 17.5% to 3.5% respectively for 10–11-year-olds since 2006/2007 [1]. Living with severe obesity in childhood has been linked to significant negative mental and physical comorbidities and complications. These include type 2 diabetes, high blood pressure, cardiovascular disease, sleep apnoea and increased mental health difficulties such as anxiety, depression, and poor quality of life, which impact on educational attainment [2–4]. If left unmanaged, these comorbidities continue into adulthood, leading to long-term impacts on quality of life and health outcomes [5]. Additionally, children and young people (CYP) living in the most deprived areas of England, as well as those identifying as black or minority ethnic populations, and those with neurodevelopmental disorders such as autism and learning difficulties, are more likely to be living with severe obesity, leading to poorer health outcomes [4, 6]. This prevalence gap has widened over time, and currently children living in the most deprived areas of the country are four times as likely to be living with severe obesity than those in the least deprived areas [1, 7]. Despite high and increasing levels of need, historically, there has been limited specialist support for the complex management for CYP living with severe obesity and associated comorbidities in England [8]. Where services do exist, there is often limited evidence of their effectiveness [8, 9]. Pre-2021, most weight management services for CYP living in England operated at the community-based level, previously referred to as ‘Tier 2’ weight management services [10]. What therefore remained was a stark and significant gap in service provision for those living with severe obesity [10, 11].

In 2019, the NHS England set the ambition to provide specialist (previously referred to as ‘Tier 3’ weight management) support for 1000 young people per year who live with severe and complex obesity [12]. As a result, NHS England commissioned a Complications of Excess Weight (CEW) programme. Twenty-one pilot CEW services were established across England in 2021, increasing to 39 services in 2024 [13]. The CEW services are delivered by multi-disciplinary teams linked to specialist children’s hospitals and aim to deliver holistic support to CYP, aged 2–18 years old, living with severe or complex forms of obesity [13]. Using different models of care tailored to local population needs and inequalities, CEW services seek to identify the factors involved in the development of severe obesity and treat associated complications [4]. Funding for CEW is ring-fenced until 2027, meaning the funding and budget is secured and specifically allocated to support the delivery of the CEW services only.

In 2024, the National Institute for Health and Care Research (NIHR) commissioned a three-year evaluation of the CEW programme, the ENHANCE (*E*valuating the *NH*S Engl*an*d *C*omplications of *E*xcess *W*eight services for CYP) project (NIHR 158453) [14]. ENHANCE adopts a realist stance to understand what type of support is most likely to work, for whom, and how the support works, and in what circumstances [15]. Specifically, ENHANCE aims to examine the design and delivery of the 39 CEW services, to understand the experiences of children, young people, and their families attending CEW, and to determine the effectiveness and cost-effectiveness of the national programme. Therefore, to develop preliminary theories about what works, it was necessary to investigate the context and aims for establishing the CEW programme [16].

The study presented in this paper sits within the wider ENHANCE project and seeks to provide the contextual backdrop to CEW commissioning, and its links to national policy. It aims to gather the perspectives of senior professionals who were involved in the design, development and commissioning of the CEW programme, to understand the intended purpose of the CEW programme, expectations around delivery, and anticipated outcomes. This is in addition to collating reflections on barriers and facilitators to the CEW programme development.

## Methods

### Design and setting

A series of semi-structured interviews were conducted with key national stakeholders to examine the design, development, and commissioning of the CEW programme in England. Ethical approvals were provided by Leeds Beckett University Ethics Committee (ref: 149049) and Sheffield Hallam University Ethics Committee (ref: 79269135).

### Sampling

Purposive sampling was utilised to identify key stakeholders who were influential in setting up the national CEW programme. As such, we identified senior officials within: (1) NHS England (the lead organisation accountable for the CEW programme), (2) the Department for Health and Social Care (DHSC; the government department to which NHS England is accountable), (3) the Office for Health Improvement and Disparities (OHID; formerly Public Health England, a directorate within DHSC, responsible for national health improvement), (4) a young people’s charity (who brought the lived experience of young people into the CEW programme design), and (5) selected senior clinicians with experience in specialist weight management for CYP who inputted into the development of the national CEW programme. We also utilised snowball sampling so that interviewees could help identify other stakeholders who were central to the CEW programme development. Fifteen stakeholders were invited to interview, 13 responded, with 11 interviewed (two unavailable). Participant identifiers (e.g. PC1) reflect the wider pool of individuals invited to take part in the study.

### Data collection

Semi-structured interviews were conducted between April and November 2024 using Microsoft Teams, lasting 30–60 min. All interviewees provided written and verbal informed consent. Together with the wider ENHANCE research team, we developed a topic guide structured around the research aims (Supplementary file 1). As such, our lines of inquiry examined: (1) Stakeholder involvement in CEW programme development, (2) The contextual background leading to programme development, (3) The intended purpose of the programme, (4) Stakeholder expectations of the programme, (5) Evaluation expectations, and (6) Future directions for the programme. The sequencing of questions, and use of probes, were flexible and tailored to the interviewee dependent on their areas of expertise. The interviews were facilitated by an experienced qualitative researcher (KK, TB, PD, or JN), who debriefed weekly to ensure the research team developed a holistic and deep understanding around the CEW programme development. Interview recordings were initially transcribed verbatim using the MS Teams auto‑transcription feature, which was then refined by a university‑approved human transcriber at Leeds Beckett University. Further details are provided in line with the COREQ guidelines [17] – see Supplementary file 2.

### Analysis

We drew upon Braun and Clarke’s methodology to undertake a pragmatic thematic analysis of the interview data, tailored for an applied evaluation context [18, 19]. The analysis was undertaken in two stages. Firstly, transcripts were shared across the research team (KC, EI, LN, MG, CH, and JN) for data familiarisation – approximately 2–3 transcripts per researcher. Researchers independently used inductive line-by-line coding on their respective transcripts and identified high-level themes. An example of a preliminary coding framework is provided in Supplementary file 4a. The research team came together to discuss and compare their individual coding frameworks and agree high-level themes and a joint coding framework. In the second stage, two researchers (EI and KC) independently applied this coding framework to half of the transcripts each (Supplementary file 4b). Following this, three researchers (EI, KC, and JN) discussed the second stage of coding and agreed a provisional set of themes, discussed and finalised with other co-authors.

## Results

### Overview of participants

Participants represented national policy- and decision- makers (e.g., NHS England, OHID, DHSC *n* = 7), established specialist weight management clinicians (*n* = 3) and a national charity (*n* = 1). Participants recognised their role in shaping the CEW programme, for example through scoping existing evidence and highlighting the gap and need for CEW. Some participants had experience leading childhood obesity services prior to the CEW programme being set up, providing valuable insights into CEW development through their clinical expertise. Others had expertise in, and supported the financial aspects, including securing the funding for CEW, the service bidding process and funding allocations for services. Aside from these roles, participants further described being involved in developing the service specification and overseeing operational aspects of CEW delivery. Quotations are labelled neutrally as PC1, PC2, etc., to protect participant anonymity; where relevant, broad professional descriptors (e.g., policymaker, clinician) are used in the narrative to support analytic interpretation, without linking roles to individual participants.

### Thematic analysis

Participants discussed the reasons for establishing the CEW pilot programme and aims of the service, in addition to reflecting on the development and perspectives of the CEW service models in practice. They further discussed both the challenges and supportive factors in developing and establishing the pilot CEW programme. Table 1 provides an overview of the main themes identified. See Supplementary file 3 for further quotes relating to each theme.

**Table 1:** Overview and summary of themes developed from interviews

Theme	Brief summary of theme content
*Reasons for developing the national CEW programme*	To address shortcomings in weight management and the lack of existing evidence for a vulnerable cohort with unmet needs, supporting the ‘long-term plan’.
*Aims of the CEW services*	To demonstrate cost-effectiveness and generate long-term evidence. Aside from weight management, aims included the use of a holistic approach to manage complications associated with excess weight, improve mental health and quality of life.
*Development of the CEW service models*	Services were established as a hub and spoke type model, where specialist hospitals could support community spokes. The specification for CEW clinics was high-level to allow for flexibility and adaptability in clinic design to foster local ownership and generate evidence. The key specification was to follow a multidisciplinary biopsychosocial approach. The importance of ongoing public involvement was recognised to ensure CEW would reflect the needs of those accessing it, given past failures in childhood obesity investments.
*Perspectives on Delivery Models in Practice*	CEW service implementation faced delays due to Trust finance processes. There were variations in the delivery models, including differential access to GLP-1s and regional oversight, however the biopsychosocial multidisciplinary ethos remained consistent.
*Challenges experienced when developing and implementing initial pilot stages of the CEW programme*	Aside from the complexity of the patient group, most challenges were largely systemic issues in the wider weight management landscape e.g. historic underfunding, fragmented service pathways, short-term planning and inconsistent infrastructure. These factors impacted the availability of the workforce. Participants also felt these factors would impact future access, especially as funding transitioned to local commissioning bodies.
*Supportive factors in the development and implementation of the CEW programme*	Contextually, alignment with the NHS long term plan and national policy commitments was supportive. Other supportive factors included the passion and dedication of service staff on the ground, increasing demand for the CEW services, and the need to build the evidence base for specialist weight management services for children

CEW, Complications of Excess Weight; NHS, National Health Service; ICB, Integrated Care Board; GLP-1 Glucagon-Like Peptide-1; MDT, Multi-Disciplinary Team

### Reasons for developing the national CEW programme

Participants recognised the CEW programme was developed to address previous shortcomings in the weight management landscape for a vulnerable cohort with unmet needs. They further recognised how this would support delivering on “the [NHS] Long Term Plan commitment” PC1, and were clear CEW was addressing a current gap in service provision:

I think it’s been set up to meet the NHS obligation for providing that Tier 3 [specialist] service for CYP with excess weight…to meet a gap in service provision that existed previously when it comes to Tier 3 services…it isn’t around…primary prevention…as opposed to the local authority Tier 2 [community-based] services… it is very much that secondary prevention Tier 3 service. PC10

In addition to addressing gaps in the current evidence base:

…despite lots of money being put in from government and childhood obesity services, we still don’t know what works, and actually obesity is going up, not down. So they haven’t been too successful. So we wanted to set up a programme that was different than we’ve done before. So that’s where the evidence generation data-led approach came from. PC2

Additionally, participants highlighted CEW would address the lack of evidence of long-term effectiveness of weight management programmes for children:

I think the original proposal was for seven pilot centres, one in each of the NHS regions, to look at services, pilot different service models to look at what worked, what didn’t work, that would then inform NHS commissioning. PC17

### Aims of the CEW services

National policy professionals and clinicians agreed that an aim of the CEW programme clinics was to generate long-term evidence and demonstrate the cost-effectiveness of services.

I would say cost effectiveness is the biggest thing we’re gonna be asked on because we know we’re a limited resource environment. Are we able to demonstrate that CEW is saving money down the line? PC10

Aside from this, policymakers and clinicians had slightly different views on the aims of the services. Policymakers generally described how the CEW services aimed to address more than just weight, but also about reversing complications, mental health and quality of life – taking a holistic approach:

When we were moving from like those 13–14 clinics to…now 21 plus…so kind of like the second iteration of it that looked at trialling non-clinical models a lot more. And that was based off of the kind of success of the psychological elements of CEW and the feedback that we were getting and understanding that a lot of the things that were coming up within like clinic was those like non-clinical needs. PC8

They also described an aim relating to targets for number of patients seen, to be able to articulate the continued need and demand for the programme. However, one policymaker described how, if they were roughly meeting that target, the efficacy of the services in terms of reversing complications was more important than numbers seen – quality over quantity. Clinicians discussed weight management as the primary focus to achieve better management of complications and other outcomes around mental health and quality of life, and felt that local commissioners would predominantly be focused on weight when commissioning these services in the future:

One is to improve young people’s health, and it is, called complications of excess weight so realistically you want to be addressing those complications… And alongside that for a lot of them is improving their engagement in education cause quite a lot of the more severely affected children are not going to school, they’ve dropped out of school PC17

…we had a national target, and I think as long as we’re like roughly meeting that target…And the main thing we’re interested in is do some clinics help more patients than others in terms of like reversing their complications? And I think that is probably more interesting than numbers in terms of targets. PC2

### Development of the CEW service models

Policymakers described the development of a very high-level specification determining how CEW services should run (e.g., a biopsychosocial approach rather than specific details). For example, information on multidisciplinary teams (MDT) focused on the functions of the MDT, considering issues like workforce availability:

There were like different example MDT configurations…within the framework document. But…the clinics were able to sort of design what that would look like. I mean, I think most of them would have, as you would expect, a consultant, a dietician, a psychologist embedded within there. But there wasn’t a sort of a strict stipulation as to what that would look like or what the whole-time equivalents would be for those roles. PC10

Several reasons were given for keeping the specification high-level, including: (1) A lack of evidence or consensus on the best model to adopt, (2) To give local services ownership with a view to creating a more effective and sustainable approach, and finally (3) CEW is an evidence generating programme.

But also wanted clinics to feel ownership over their clinic and if you give a very specific spec of you must do it like this… it wouldn’t feel like their thing. And then again, it’s harder to, I guess there’s just less local pride in your clinic if someone told you exactly how to do it and you have less ownership over it. So, this is just how it’d be the easiest way to create a more sustainable service that people want to work in and are bought into… we would have had no basis to be so specific. There is no evidence base really on like what you must provide… the combination of bringing together mental health, kind of social context, with acute physical complications for this group doesn’t have a very strong evidence base on how you do it. (PC2)

However, one clinician expressed that specific aspects, such as patient resources, would benefit from coordination nationally.

a lot of this could be coordinated and be done at a national level…information to be shared with patients…even in terms of dietary approach or what activities they could do, some videos to watch, should this all be not set up at a national level? PC7

Policymakers further described how CEW services were set up as a hub and spoke model with ‘hubs’ linked to specialist children’s hospitals and with ‘spoke’ sites based in non-specialist centres supporting the hubs. Spoke services were perceived to be cheaper, and were set up to increase equitable access to CEW clinics across the country:

We have like a hub and spoke / networked model across the country. What we set up in the first round was the hub infrastructure, which is linked to specialist children’s hospitals that have all the specialties on site you could possibly need to treat this cohort. Along with that kind of first round of clinics, we also had some split sites and some kind of spoke supporting the hubs depending on geography and spread. And then this kind of last round of expansion was to make sure that every ICS across the country had access to a service. PC2

Professional networks, for example, for dietitians and specialist nurses working in CEW teams were also established as a key part of the programme to share lessons learned between services and regions.

Finally, participants discussed the benefits of the initial engagement work undertaken with children, young people and families with lived experience of severe obesity to develop the CEW programme. This was undertaken by skilled facilitators from an external organisation and informed the service specification.

…they helped to bring like young people’s voices… And they had a lot of input on the mental health elements because I think that came out quite strongly in terms of what was important to measure. PC8

Participants, however, highlighted the need for public involvement at the service level as well as the programme level:

I think that people do seem to recognise in CEW clinics the importance of the engagement stuff…. They’ve really valued it…they found it really helpful for their development of their clinics…But I don’t think that…it’s fully embedded in each of the, in CEW, and I don’t think the value of engagement has been fully understood in terms of how it can support young people and families weight loss. Basically, how it can support the main aims of CEW…because there’s very much a clinical approach. PC12

### Perspectives on delivery models in practice

Both policymakers and clinicians noted that the numbers of CYP accessing the services and on the waiting, lists were increasing since the service inception. However, while not the case in all regions, the implementation of CEW was slower and less resilient than expected due to local financial processes:

When you allocate money to a [NHS] Trust, a Trust will still have to go through their own business planning processes and, yeah, just the time it takes for a Trust to do business planning, HR, procurement, all of that, has been really eye opening… a lot of Trusts still need yearly confirmation of budget PC2

Participants felt, however, that the core principle of the biopsychosocial MDT approach of services were being adhered to. This was based on their own observations and feedback from CEW teams, CYP and their families. The prescribing of GLP-1 receptor agonist medications (e.g. semaglutide) varied between CEW services, given that there is no national clinical guidance in place for their uptake in CYP. Therefore, the decision to fund GLP-1s is made at the local level.

So prescribing costs…that comes out from a different budget within the NHS and that is something that I think we are hearing more about from clinics is around GLP-1 agonists and prescribing. And there is variation across the CEW clinics because it very much depends on whether they’ve been able to get agreement from either their ICB [Integrated Care Board] or their trust division or budgets, medical, prescribing budgets to use GLP-1s… PC10

Whilst clinicians recognised their services were helping CYP, they were unsure whether the care they provided was the most effective compared to other services. This in part, informed the decision to commission the national evaluation:

You always like to think that your service is the best, but everyone thinks theirs is, don’t they? So that, you know, it’ll, it’ll come out in the wash won’t it really, how many have you seen? Are you proportionately more expensive? Are your pounds per kilo higher than everyone else’s? Are your pounds per self-esteem increased points score, more efficient, more effective? PC17

### Challenges experienced when developing and implementing initial pilot stages of the CEW programme

The challenges regarding the pilot and CEW services were primarily related to wider contextual issues that have ‘knock on’ effects. Examples included the lack of historic funding in weight management which fuels ‘short-term’ thinking about the service, and the overall fragmented wrap around service provision due to a lack of infrastructure and pathways, which varies by region:

…we talk about like in a step-down approach or step-up approach from prevention to Tier 2 weight management programmes then escalating to Tier 3. That is non-existent. The Tier 2 is non-existent in many, many regions, you know. That’s hit and miss and it’s so variable. PC7

Participants discussed the growing numbers of CYP living with obesity and associated complications highlighting the need for continued investment, whilst foregrounding the challenges of a short-term pilot:

…. we are still fighting and we know that services are needed, without a doubt there is a need, but it’s trying to make the case for investing, you know, in return on investment. And it depends on the government in power. PC3

Going further, the consistency of contracting and subsequent loss of staff, staff turnover and national workforce shortages were discussed, alongside the ability to address the increasingly high demand for the service amongst worries of a lack of adequate future funding, given the future need to transition to local (ICB) funding:

We’ve been able to centrally fund CEW from our programme budget this financial year. We’re aiming to make the same argument to be able to fund CEW centrally next financial year as well, but the expectation is that at some point it has to go into ICB baselines and ICBs are the ones that then make the commissioning decisions as to whether they want to continue funding CEW PC10

However, whilst CEW has been commissioned as a pilot programme, many participants highlighted that it was not perceived as such by all, which could lead to unrealistic expectations around national availability and sustainability of the services.

…this approach about it being a really long pilot in terms of the development I think is still quite misunderstood, so people think we have a Tier 3 service for all children and young people, but that’s never really been the ambition. Our ambition is to develop a service model that works. Find out the good bits, the bad bits, what patients like. And then at the end of the programme, be able to recommend a model of care for kind of proper commissioning. So, we do get quite a lot of pushbacks around kind of lack of access…But that is expected in a pilot programme. You can’t go big and provide a care offer for the 1.2 million children, when you don’t know what works at the at the start. PC2

Policymakers discussed the need to ‘act fast’, despite requiring time to establish the services. This was influenced by: (1) the impact of the COVID-19 pandemic on service set up, (2) the lack of existing evidence or services from which to base the service design and (3) the complexity (both familial and contextual) of the population who require the CEW services:

If we we’re successful with 50% of the families we deal with that would be a world leading service… because a lot of the key determinants are not within our gift to change, you know, grinding poverty, parents got mental health problems. You can advocate and try and address those as much as we’re able, but there are some households where we’re just sadly not able to do that. PC17

### Supportive factors in the development and implementation of the CEW programme

Contextual supportive factors discussed, which participants felt should facilitate future funding, included (1) The NHS Long Term Plan (now replaced by the NHS 10 Year Plan), (2) The national policy commitment to support CYP living with obesity and (3) The growing waiting lists being indicative of service demand.

The passion of CEW staff was noted as a key driver and success to the CEW service delivery:

This is a really a fantastic development. We were very excited when the services came because we’ve been waiting for 20 years or something for obesity services for children, and were absolutely delighted at this move towards setting up these services came - and we are really in a good position now, set up these services, trained people, MDT are hardworking and the people are passionate, want to making a change PC7

Additionally, the networking and sharing of learning across services was often seen as important to the strength and success of CEW services, despite one participant noting that enthusiasm for these networks had waned in more recent times. Finally, participants felt the creation of a quality data collection system would: (1) Evidence the need for the CEW programme to support obtaining future funding, and (2) Build on the evidence base as one of the goals in establishing the CEW programme.

We need a number of parameters to show that our service is worthwhile and we are showing some, demonstrating some improvements on outcome PC7

## Discussion

This paper presents the decision-making processes from key stakeholders involved in the initiation, set up and delivery of the CEW services in England. It provides a unique and original overview of the considerations which lead to the roll out of these specialist health services for children, young people and their families and provides learning, reflections and recommendations which can be adopted for future paediatric health service design. Seven overarching themes were identified from the data which highlight the role of stakeholders in the development of the service and important contribution of meaningful public involvement, the perspectives on delivery, the challenges that the CEW services face and the factors which facilitate and support the delivery and roll out of the services.

This work recognises the complexity of the health and care landscape and its interconnectedness with political and wider systems over which services have little control or impact. Historic underfunding of NHS weight management provision, limited consideration for the wider obesogenic environment and systems in which they sit, and the repetitive failure of obesity policy were all discussed as challenges within the service delivery remit [20]. CEW services are currently compounded by short-term ring-fenced funding which limits their ability to integrate with the wider weight management- and health and social care- services. The short-term funding is problematic for staff recruitment and retention and is a known barrier to patient trust and engagement [21]. Therefore, given the need and work to establish CEW services, local commissioning bodies must be engaged in conversations to support the continued provision of CEW and wider roll out beyond the pilot areas.

Overwhelmingly, participants recognised the importance of the biopsychosocial model of health and the need to consider, and act on, the wider determinants of health in which CYP and families are living. Whilst this was understood strongly by national policymakers and commissioners, there was a tension between this, and a very biomedical stance taken by some clinicians who were deemed to have more power and scope to ‘do their own thing’ when it came to service design and set up. Consultation with public and patients also highlighted the complexity of obesity [22], however, more work is required to ensure their voices and experience are truly valued and embedded in a more sustained way within the CEW services. Despite using an expert independent body and early Patient and Public Involvement and Engagement (PPIE) in CEW design, interview participants still felt there could be better incorporation of the needs and wants of CYP living with obesity. Therefore, even with the production of toolkits to strengthen PPIE [23], the voices of those with lived experience may still be overlooked when evaluating and commissioning important and impactful services. For example, participants noted that decision‑makers often prioritise measurable clinical indicators and cost‑effectiveness, which may mean that broader outcomes such as quality‑of‑life improvements receive comparatively less attention, despite being viewed by some with lived experience as important markers of success. Given the complexity of obesity [24], and the potential impact of outcomes that are not strictly weight related, it is important to work towards shifting the importance placed on weight measures alone [25].

Participants described their broad and varied roles in the development of CEW services and discussed the lack of existing evidence for working in such complexity. They further highlighted the disconnect between policy and practice alignment in some areas, such as the aims of the CEW services to treat weight predominantly versus a broader set of holistic outcomes, the realities of local funding and procurement processes required to get a new service up and running, and the misunderstanding of what could be achieved within a pilot programme. These findings are not unique to CEW, with well-intended policies having previously been recognised for overlooking complexity, impacting intended outcomes [26]. Therefore, this could explain why there were some differences between what was expected to be achieved by the CEW services, and the reality of what was delivered. The need to balance ‘top-down’ deployment with ‘bottom-up’ discovery is needed to align policies with practical realties [27]. Therefore, going forward, feedback from services regarding the challenges and limitations must be considered, with support for overcoming these within future policy iterations and service specifications.

Service leads were expected to develop CEW provision in line with a high‑level service specification [13], which acted as guidance for piloting the programme and generating evidence to inform a future service framework. Prior to the setup of these services, there was a paucity of evidence and guidelines about what a specialist weight management service for children should look like [28]. Therefore, there was a strong sense that the CEW services were an opportunity to pilot new ideas and health service models in an attempt to deliver and expand the evidence base. Given a frequently reported barrier to sustainability is inadequate staff resourcing [29], this flexibility could be helpful for supporting CEW longevity within regions. It has also been acknowledged that ‘enhanced local autonomy’ can make services more resilient [30], for example, sites and services evolve over time due to the demand on services. However, participants further emphasised the need for more national input, at the very least in terms of shared evidence-based resources to relieve pressure on local resources. This could also be an important consideration to reduce the potential for weight stigma amongst health professionals impacting quality of care [31, 32]. Therefore, it is a balancing act between allowing some flexibility to support longevity and resilience of services, whilst relieving local pressures and ensuring biases do not take over through lack of adequate national input.

### Strengths and limitations

This is the first in a series of studies which highlights the evolution of the CEW services in England as part of the ENHANCE research project. This study describes the role of stakeholders and public members in contributing to the design and setup of the CEW weight management services. Eleven out of 15 invited participants took part in the interviews which provided valuable insights and recommendations from which other health care commissioners can adopt best practice when developing services in a field which is evidence-poor.

This study was limited by the small number of stakeholders who were involved in the development of CEW services led by NHS England, which led to limited perspectives from highly vested individuals. Due to the politically sensitive nature of some of the interview topics, participants may have been more guarded in sharing information with the research team.

### Implications for practice

- Commissioning of services benefits from investment in Patient and Public Involvement and Engagement and a diverse range of stakeholder voices which should be embedded throughout service development, implementation and evaluation.
- Long term and sustained investment help to provide suitable services which deliver for underserved and complex population groups.
- Understanding nuances and differences between areas and in the needs of CYP can help appropriately tailor services to community needs.
- Staffing challenges from short-term contracts need addressing to ensure recruitment and retention of suitably qualified and well-motivated staff.
- Holistic, socioenvironmental approaches are deemed important by policy leads and public members, but further work is needed to improve clinician and local commissioning buy-in to this approach to improve consistency across services. clinicians.

## Conclusions

Overall, this study has presented insights into the development of CEW services from a range of key stakeholders, highlighting the importance of public involvement in service design and evaluation. The context within which CEW is delivered must be considered, including fragmentation and funding across the system. There is a need for sustained investment in CEW, which incorporates a holistic MDT design to ensure equitable and effective healthcare provision for this complex and vulnerable patient group.

## Electronic Supplementary Material

Below is the link to the electronic supplementary material.

Supplementary Material 1

Supplementary Material 2

Supplementary Material 3

Supplementary Material 4

Supplementary Material 5

## Data Availability

The datasets used and/or analysed during the current study are available from the corresponding author on reasonable request.

## Abbreviations

CEW: Complications of Excess Weight
PPIE: Patient and Public Involvement and Engagement
CYP: Children and Young People
ENHANCE: Evaluations of the NHS Complications of Excess Weight clinics for Children and Young People
BMI: Body Mass Index
NHS: National Health Service
GLP-1: Glucagon-Like Peptide-1
COVID-19: Coronavirus disease
MDT: Multi-Disciplinary Team
ADHD: Attention Deficit Hyperactivity Disorder
ASD: Autism Spectrum Disorder
ICB: Integrated Care Board
