# Supplementary material for: From policy to person-centred care: insights into the development of complications of excess weight clinics in England
Source: BMC Health Serv Res. 2026 May 13;26:849. doi: 10.1186/s12913-026-14689-7 (PMC13281504; doi:10.1186/s12913-026-14689-7)
Supplement: Supplementary file 1 — Supplementary Material 1 [file 12913_2026_14689_MOESM1_ESM.docx]

**Additional file 1 - Topic guide**

| **1.** | **Involvement** \| How have you been involved in the development of the CEW Services programme over the last few years? |
| --- | --- |
| **2.** | **Background to the services** \| Could you talk me through some of the background which led to the idea of the CEW services coming about and then being developed?   - *Probe*: Where did the idea for the services originate? - *Probe*: How long did it take for the CEW idea to be developed into a programme? - *Probe*: What supported or hindered the development of the CEW service programme? - *Probe*: Who was consulted with as part of this process?   - People with a lived experience of severe / complex obesity? |
| **3.** | **Intended purpose** \| When designing the CEW services programme, what have they been set up to try and achieve?   - *Probe*: Thinking about this in terms of the short, medium, and long term. - *Probe*: Do the CEW services align with any broader policies beyond those included in the NHS Long Term Plan? - *Probe*: What do you hope that is achieved via the CEW service programme (within the evaluation period of 32 months)? - *Probe*: Do you think the vision for their intended purpose is shared by all? |
| **4.** | **Expected delivery models** \| What do you expect that the local CEW service models will look like?   - *Probe*: How does the early implementation of the CEW services compare to what you expected in the policy design phase? - *Probe*: What was the rationale for encouraging local areas to design their own approach to CEW delivery rather than imposing a more top-down structure? - *Probe*: Were there any stipulations as to how they should operate?   - What about the workforce models in terms of the staff who are involved in their design and delivery?   - Are there any expectations about the type of content or interventions that the CEW services should include? (*Are they expected to differ significantly from traditional delivery models?*)   *Probe*: How do you anticipate the new CEW services will learn from, or differ from, the original 21 services?   - *Probe*: Has there been much variation in terms of the cost of delivering the service using different delivery models? For example,   - Are you expecting differences in the cost of delivering the service in community settings rather than hospitals or with different mix of healthcare professionals?   - What lessons can be learnt about the cost-efficient delivery of these services in future?   - What do you think are the main drivers of cost differences between sites?   - What was (your perception) of the impact of different delivery models on outcomes, including reaching people that are hardest to reach? |
| **5.** | **Evaluation expectations** \| What are your thoughts regarding the national evaluation of the CEW services?   - *Probe*: What are you hoping to learn from the evaluation? - *Probe*: How do you see the evaluation influencing the future commissioning of the CEW services? - *Probe*: Do you have any hesitations surrounding the capabilities of the evaluation? (i.e. whether certain aspects are more or less feasible) |
| **6.** | **Future hopes \|** Where do you hope that this work goes in the future?   - Prompt: How do you hope that the CEW services or the wider policy context will evolve in the future? |
| **7.** | **Anything else** \| Recap- what are the CEW trying to achieve overall- the most important outcomes (e.g. BMI, QoL, improved wellbeing etc)  Is there anything else that you would like to mention regarding the CEW service programme and its evaluation? |
| **8.** | **Snowballing** \| Do you have any recommendations on who else might be useful to speak with about the planning, implementation, or monitoring of this policy?  Anyone involved in the background, initial conversations about broader policy. |
