# Supplementary material for: From policy to person-centred care: insights into the development of complications of excess weight clinics in England
Source: BMC Health Serv Res. 2026 May 13;26:849. doi: 10.1186/s12913-026-14689-7 (PMC13281504; doi:10.1186/s12913-026-14689-7)
Supplement: Supplementary file 2 — Supplementary Material 2 [file 12913_2026_14689_MOESM2_ESM.docx]

**Additional file 2 - COREQ Guidelines**

| **COREQ item** | **Response** |
| --- | --- |
| ***Domain 1: Research team and reflexivity*** | |
| Interviewer(s) | Interviewers included:  Karina Kinsella (KK)  Dr Tamara Brown (TB)  Paige Davies (PD)  Dr James Nobles (JN) |
| Credentials and occupation | KK: Research Assistant  TB: Reader of Public Health (PhD)  PD: Research Assistant  JN: Reader of Public Health (PhD) |
| Gender | KK: Female  TB: Female  PD: Female  JN: Male |
| Experience and training | All interviewers have extensive experience (>5 years) in conducting qualitative research. TB, PD, and JN have PhD qualifications (or working towards a PhD) which draw upon the use of qualitative methods. |
| Relationship with participants | The research team did not have any pre-existing relationships with those that were interviewed. Some participants work closely with the ENHANCE project team, and do have professional relationships with other ENHANCE project members, but care was taken when pairing researchers and participants to ensure that a pre-existing relationship did not influence data collection. |
| Participant knowledge of interviewer | Participants were made aware as to the objectives of the research study, including how the study fits within the broader ENHANCE project. Participants were not made aware though of the personal goals and motivations of the researchers. All researchers were funded, as a proportion of their full time equivalent, to work on ENHANCE. |
| Interviewer characteristics | Participants were not made aware about researcher assumptions, reasons, or interests in the research topic. |
| ***Domain 2: Study design*** | |
| Methodological orientation | This study was a pragmatic qualitative exploration of key informant perceptions regarding the development of the national CEW programme. |
| Sampling | Critical case- and snowball- sampling were used to identify prospective interviewees. |
| Method of approach | Prospective interviewees were invited to participate via email. |
| Sample size | We aimed for a sample of 10 interviewees. Rationale was based upon our pre-existing knowledge of the policy development process, and who was involved. We left room for additional stakeholders to be identified via snowball sampling. |
| Non-participation | 15 stakeholders were invited to interview, 13 responded, and 11 were interviewed. Of the two who responded and did not interview, one was unavailable during the data collection window and we were unable to secure an interview with the second, despite repeated attempts. We sent two follow up reminders to those who did not respond at all. |
| Setting of data collection | All interviews were completed online using the Microsoft Teams video conferencing software, using a Leeds Beckett University organisational licence. |
| Presence of non-participants | There were no non-participants present in any of the interviews. |
| Description of sample | Sample characteristics are reported in the results of the paper. |
| Interview guide | The interview guide development is described in the methodology, and guide itself is included as an online supplement. |
| Repeat interviews | No repeat interviews were carried out. |
| Audio / visual recording | Interviews were recorded using the Microsoft Teams functionality as an MP4 file. The automated transcript from Microsoft Teams was used to support the outsourced transcription. |
| Field notes | Notes were made by the research team during interviews, but solely for their own benefit to help guide subsequent follow up questions. Field notes were not analysed within the data corpus. |
| Duration | Interviews ranged from 30-60 minutes. |
| Data saturation | We did not aim to reach data saturation. |
| Transcripts returned | Transcripts were not returned to participants. |
| ***Domain 3: Analysis and findings*** | |
| Number of data coders | The full coding process is described in the methodology. |
| Description of coding tree | Our initial coding framework, used in the first stage of analysis, was guided by our topic guide. This was subsequently refined after the first stage of analysis was completed by the six researchers. The coding tree therefore included high-level categories broadly pertaining to the topic guide, with the development of minor themes within these categories. This coding tree was refined by the research team prior to the second stage of analysis by the two researchers (EI and KC). |
| Derivation of themes | Following final and full coding by EI and KC, the two researchers met with JN to discuss major and minor themes, and identify overlap and difference. Final major and minor themes were agreed upon through discussion between the three researchers. |
| Software | NVivo was used to support data management. |
| Participant checking | Participants were not consulted throughout, or following, the data analysis. |
| Quotations presented | Participant quotations are included in the results with participant numbers used to support quote identification. |
| Data and findings consistent | There is consistency between the data presented and the findings, and this was something that was checked by the research team as part of the analysis. |
| Clarity of major themes | Major themes are clearly presented in the results – see relevant section of the paper. |
| Clarity of minor themes | Minor themes are clearly presented in the results – see relevant section of the paper. |
