# Supplementary material for: From policy to person-centred care: insights into the development of complications of excess weight clinics in England
Source: BMC Health Serv Res. 2026 May 13;26:849. doi: 10.1186/s12913-026-14689-7 (PMC13281504; doi:10.1186/s12913-026-14689-7)
Supplement: Supplementary file 3 — Supplementary Material 3 [file 12913_2026_14689_MOESM3_ESM.docx]

**Additional file 3**

Additional quotes from the results, removed from the main text for conciseness and wordcount. They emphasise or align with points addressed in the manuscript and are organised according to the theme they demonstrate and/or align with.

***Reasons for developing the national CEW programme***

“I think from a policy perspective, we were always interested in whether the service works over a longer period of time, since a lot of weight management type of programmes have trouble indicating benefit over a long period of time. So that is our kind of key policy problem…we also had the National Audit Office wrote a report on the state of childhood obesity services in general…And that basically said that despite lots of money being put in from government and childhood obesity services, we still don't know what works, and actually obesity is going up, not down. So they haven't been too successful. So we wanted to set up a programme that was different than we've done before. So that's where the evidence generation data-led approach came from.” PC2

“... in the longer term the learning from it, the data, the links into the wider services might support us with coming up with interventions that are going to help prevent people being in acute clinic in the first place. So that was our sort of argument back, and it might help us build the business case for other programmes of work nationally to support, to support children from being severely obese in the first place." PC4

***Aims of the CEW clinics***

“I would say cost effectiveness is the biggest thing we're gonna be asked on because we know we're a limited resource environment. Are we able to demonstrate that CEW is saving money down the line? So does the CEW service provide an avenue to be able to identify early complications which otherwise would have presented to the system much later and would have cost the NHS more? I think that's kind of where if you like the value added I think comes in from the CEW.” PC10

*“…there's obviously the clinical outcomes of like sleep apnoea or trying to reduce type 2 diabetes or whatever. But there's also…PREMs, PROMs…to try and also measure people’s quality of life and how happy they are, mental health. I think central to our outcomes we want to achieve, was a mixture of that…what are the quality of life of these CYP as well as the clinical outcomes.”* PC4

“…we had a national target, and I think as long as we're like roughly meeting that target…And the main thing we're interested in is do some clinics help more patients than others in terms of like reversing their complications? And I think that is probably more interesting than numbers in terms of targets.” PC2

*“I think the original proposal was for seven pilot centres, one in each of the NHS regions, to look at services, pilot different service models to look at what worked, what didn’t work, that would then inform NHS commissioning.”* PC17

“It's a weight management clinic for people with complications of excess weight. Thus, if they’ve got complications of excess weight, they need to lose weight. That is the purpose of the clinic. And I make no bones about that. You can have lots of secondary outcomes like in reducing stigmatisation, improving self-esteem. These will inevitably result from losing weight because young people feel so much better when they've lost weight, are more mobile, their complications have gone away. They have to be outcomes that relate to the complications which are due to excess weight, so weight reduction has to be at the core of what the outcomes should be” PC16

***Overview of the public involvement/engagement work that informed CEW***

“…the proper co-development of the service is still ongoing…So this is all undergoing a quite a long four to five year pilot period where at the end we want to have a service specification commissioning spec that says this is what a CEW service is, this is what we know works and doesn't work, this is what patients think, and here's the data to show that it works. So I'd say we're not really there yet, but patient engagement has underpinned the whole programme.” PC2

“...they helped to bring like young people's voices...And they had a lot of input on the mental health elements because I think that came out quite strongly in terms of what was important to measure. And it really did help to steer, the final metrics because I think it was sort of like physical health heavy at first, but it just reinforced that...there needs to be like an equal mental health element in terms of the data that we're collecting and the psychological impact that the CEW service has.” PC8

***Expected delivery models***

*“although every clinic had the autonomy to set up in the way that they wanted to, we guided it through saying you must have these essential professionals and you must be taking a biopsychosocial approach. And this needs to be very much intervention trying to address those complications, not just come in, see a doctor, get given a medication and told to go and stop eating so much and do more activity because that's kind of what's happened in traditional services.”* PC1

*“we’ve given a sort of model of the type of MDTs with it’s the staff we'd expect and…that's how we’d costed it up, you see. You know, we'd costed it up based on a bit of consultant time and mental health nurse, dietitian. So, there is like a finite resource. You couldn't just have 10 consultants and 20 dieticians. So, that's probably the limiting factor on variation because it's, we've costed it up roughly on one of the clinics that's better established.”* PC4

*“So as far as I'm aware, I don't think there was a, a clear stipulation on what the focus of the model should be. And again I think that's because part of CEW is it’s very much is an evidence generating programme, isn't it? It's a test bed in many ways.”* PC10

“We had the tension of doing it in the right way…but also the tension of we've got a target to hit and we need to get to that target. And the best way to get people to, in a way the target… if I want the tertiary centres to get behind this and to set up…and start getting throughput you've got to make sure you've given them the support to set it up the way it's going to work for them.” PC4

“there was hours and hours and hours of debate about the detail…and everyone wants an MDT that's slightly different.” PC4

“We wanted clinics to feel ownership over their clinic and if you give a very specific spec of you must do it like this… if someone told you exactly how to do it you have less ownership over it.” PC2

“…it'd be my impression that it would be cheaper within the community because the idea of like the spoke clinics is that…it's more about I suppose that clinician or like the member of the MDT interaction with that patient as opposed to having medical equipment and things like that, that might be in a bigger hospital, which might be a little bit more expensive. So yeah, it would be my impression that community and spoke clinics would be cheaper.” PC9

“…we've got things like the Evira Medtech that we're testing out again. In many respects, I think this is probably…a unique service…at least from the literature that I've seen, there isn't very many international comparisons when you have these sorts of clinics set up.” PC10

***Actual delivery models***

“So it seemed like a really great service in theory, right? But actually like going into clinics…and seeing how like people being supported and helped and just the compassion that our clinicians and the MDT has had, it's kind of exceeded, it's met and exceeded expectations.” PC8

“We had everyone in place…we had the infrastructure in place, we had the clinic times, we had the clinic rooms. We had a sort of demonstrable schema of who we were going to see. We had admission guidelines and everything like that. So it wasn't very difficult. But I'm sure lots of other places had much more difficulty.” PC16

“...the model for our service was very much based on my personal experience as a clinician and what I knew from the literature and from personal experience about what works and what doesn’t work... And also we’d done lots of public patient involvement work over the years about what young people wanted for services. We didn’t redo that specifically but...basically we took on board what families and kids had said, what was important to them” PC17

“we're multidisciplinary clinic so that evolves…I wouldn't say that the model that I set up is necessarily the model we have now because everyone's put huge amounts of effort into developing their specific disciplines…for instance looking at binge eating disorders, which are quite common amongst the young people we see, that was all set up by a psychologist and stuff. So it's an evolving process, but the basics was kind of me, what I thought would be good.” PC16

“…our regional programmes have set these regional CEW oversight groups up which they bring together all the clinics. I’d probably say where they slightly differ is in their emphasis around the wider obesity pathway. So some of them have brought in public health colleagues, OHID, into those oversight groups as well, and will be doing a bit more thinking around what that pathway looks like around step up, step down into CEW, for example. Whereas others have just taken a bit more of a focus just on the CEW services themselves. Some have brought in their ICBs because we know that's where future commissioning is going to lie, so their ICBs are part of those conversations from an early stage. I think most of them are starting to do that now actually, or the ICBs are invited, but as to whether they're engaged is depends as variable across the piece.” PC10

“there is variation in MDTs, there is variation in what goes on, but I think the principles of what they're doing is all really similar…I think all the CEW clinics have been sort of built around, right, we're here to support you holistically… in a personalised way…I think all of them have got that ethos and they might do it slightly differently and they might have different links into the community, the hubs and spokes and things. But yeah, I think, consistently they're all working that way.” PC4

“So initially the thought was actually having a tertiary centre, a Children's Hospital, would be able to provide all the different services that they need to address all the different complications. But my feeling is watching it happen actually that's made it more difficult for some services because they are so specialist in what they deliver. Therefore the respiratory service needs to deliver this bit and the liver service needs to deliver this bit. But whereas when you look at some of the like district generals, they're used to delivering everything, so they just get on and deliver everything so the patients are actually getting a more holistic service where they're not in a tertiary centre. And also the tertiary centres are often only up to the age of 16 and that's where it becomes a problem that they can't see older children because they wouldn't be able to be admitted to that hospital therefore that trust says no, we don't see children over that age. So there are some nuances there that, you know, maybe unintended consequences if you like of thinking, oh a specialist tertiary centre is where they must get the best care. And I'm not sure that that's actually playing out to be true from that point of view.” PC1

“But actually a lot of them have merged really in the ways that they're doing things. So digital is used within most services. The digital first service in [city] actually still do a face-to-face initial appointment…they’re not that dissimilar…There are still distinct differences, but I don't know that you can kind of group them in the same way. They've come a lot closer together than their initial brief that they sent in as how they wanted to deliver was, if that makes sense” PC1

“*…part of me thinks we might be more expensive but, at the moment, we’ve got a similar budget, we’re seeing certainly in the last figures I saw from NHS England we’re seeing more patients than a lot of the other centres are, or at least as many as the other centres so I don’t think in terms of bums on seats we’re lagging behind…”* PC17

“So the numbers of type 2 diabetes going up looked after by paediatricians…it's increasing at a pace…Now there's about 1000 young people in paediatric clinics with type 2 diabetes and we've got so many now that we can actually fill type 2 diabetes clinics on a once monthly basis and see every patient every three months as per the best practice tariff for diabetes. So we're just going to see more and more people with significant complications because I'm afraid I don't believe that public health interventions have shown any effectiveness yet.” PC16

“I thought the concept was to look at what worked and what didn’t work and then commission nationally and they’ve not really done that. They’ve rolled out I’ve forgotten how many centres there is now, is there 23, 24, or something like that. So instead of having seven centres that were properly going to pilot things, what works, how to spend the money most effectively, we’ve now got everybody, everyone in the UK needs access to a Tier 3 service and we’re doing the what works, doesn’t work, the wrong way round…we’re doing it after the event rather than before the event. But that’s often how it works with NHS pilot services. I’ve been there before where they’ve run service as a pilot and they don’t wait for the results of the pilot, they roll it out and you think well why did we do a pilot ‘cause we’ve not waited or learned from it.” PC17

***Challenges experienced when developing and implementing initial pilot stages of the CEW programme***

*“There isn't anything that we have proactively done about transition, but I've been around long enough to know that that transition between adult, between children’s services and adult services, not just in obesity but across the board, is quite a tricky area.”* PC13

*“The sad thing is obesity becomes always an optional thing whether it should be funded or not and how long in this country we're going to be debating whether we need a service for it.”* PC7

*“The issues of having this as being a pilot scheme, so we kept being very mindful of how many number of patients we can support and how long and things like that... so the active number of patients in the service become more and more as time goes by. So every year we take a hundred new, but then the existing patients from previous year would also be there. So for example, we have more than, I don't know, around, around 250 active patients at the moment. So that becomes the pressure for services and the resources what we have been offered. So that's, that's one issue in terms of the numbers.”* PC7

*“How much of a priority we give to certain things depends on the ministerial team that we have... we've just had a change of government, so one of the things which we're still waiting to see is what's the priority going to be for the new government in this space? But one of the things that we do know is that, and it was all set out in manifesto commitments and stuff, is that one of their aims is to achieve the healthiest generation of children. And so, we know that tackling childhood obesity sort of is one of the things which they're going to need to do”* PC13

*“I definitely do think of all pilots, you know, sometimes recruitment, particularly for roles like your admin roles or maybe like lower banding roles, it's not as attractive because people want stability, you know and things like that. And yeah, like high pay and things so that can be quite a challenge I think, yeah, possibly with recruitment. It hasn't been a bigger challenge that it's like created, like it's been a significant risk as with like some of our other programmes, but yeah, I do believe that that, you know, kind of hindered mobilisation just a little bit”* PC8

*“This is, this was really a challenging group of patients. Now these, you're talking about teenagers mostly or children with a severe weight mostly have over than 100 kilos, with lot of mental health. Our population like 30-40% have learning difficulties or ADHD, ASD, and 50% will have mental health issues, so that is the group we are tackling. So to show outcomes in terms of 5% weight loss, 10% weight loss is really difficult.” PC7*

*“instead of having seven centres that were properly going to pilot, you know, things, what works, how to, how to, you know, spend the money most effectively, we’ve now got everybody, every, everyone in the UK needs access to a Tier 3 service and we’re doing the sort of the what works, doesn’t work, the wrong way round.”* PC17

***Supportive factors in the development of the CEW programme***

*“It came from the NHS long term plan… a National Audit, National Obesity Audit identified that there's like huge levels of paediatric obesity and that there's a gap within services. And essentially that, yeah, there's an obesity epidemic within our, within our CYP. So when the NHS long term plan was published, that was one of the big paediatric commitments within there. So yeah, CEW was established off the back of that and there was lots of I suppose organisational interests and like funding to support that development.”* PC8

“the engagement at least with what I could see from clinicians with time went down and down... if you go for some of the latest meetings, there's not really many people joining at all” PC7

“And so, for example, there's been quite a lot of like quality improvement as we've seen in action… they work together on developing resources and ways of working. So I think that's a real strength of the programme. I think these networks are really crucial to that because [clinics] have been able to learn together and change together.” PC1

*“Because I suppose it was all about, that's like the main aim of this thing to, you know, build on the evidence base.”* PC8
