# Supplementary material for: From policy to person-centred care: insights into the development of complications of excess weight clinics in England
Source: BMC Health Serv Res. 2026 May 13;26:849. doi: 10.1186/s12913-026-14689-7 (PMC13281504; doi:10.1186/s12913-026-14689-7)
Supplement: Supplementary file 4 — Supplementary Material 4 [file 12913_2026_14689_MOESM4_ESM.docx]

ENHANCE policy interviews

Codes

Codes\\1. Key contextual factors surrounding CEW clinics

| Name | Files | References |
| --- | --- | --- |
| Complexity of the CEW population | 2 | 2 |
| Lack of understanding that CEW is still a pilot programme | 2 | 2 |
| NHS long-term plan | 1 | 1 |
| Poor obesity services infrastructure and funding | 2 | 3 |
| Short-term funding and workforce challenges | 3 | 3 |
| Short-term government thinking - we are not geared up for early intervention | 1 | 1 |
| The prevention narrative | 1 | 1 |

Codes\\2. Priorities of young people consulted

| Name | Files | References |
| --- | --- | --- |
| Mental health | 1 | 1 |
| Reports to consult | 1 | 1 |

Codes\\3. Goals or aims of the CEW clinics

| Name | Files | References |
| --- | --- | --- |
| A mixture of clinical and psychosocial improvements | 2 | 2 |
| Establishing whether the services work over a longer period of time | 1 | 1 |
| Long-term plan commitment | 1 | 1 |

Codes\\4. Key features of the CEW clinics

| Name | Files | References |
| --- | --- | --- |
| Education strategy to support professionals incl networks of practice | 2 | 4 |
| Holistic, biopsychosocial approach | 3 | 4 |
| MDT approach | 3 | 6 |

Codes\\5. Reasons for not having very specific stipulations for CEW clinics

| Name | Files | References |
| --- | --- | --- |
| Financial reasons | 1 | 1 |
| It's a pilot | 1 | 1 |
| Lack of evidence base | 2 | 2 |
| Targets to hit | 2 | 3 |

Codes\\6. Referral criteria

| Name | Files | References |
| --- | --- | --- |
| Difficulties prioritising | 1 | 1 |

Codes\\7. Selection of outcomes or dataset

| Name | Files | References |
| --- | --- | --- |
| Methods used | 1 | 1 |
| Social and emotional outcomes a key focus for YP and families | 1 | 1 |

Codes\\8. Models of delivery

| Name | Files | References |
| --- | --- | --- |
| Difficult to differentiate or group services - things have changed over time | 1 | 1 |

Codes\\9. Funding or cost basis of CEW clinics

| Name | Files | References |
| --- | --- | --- |
| How costs were determined per clinic | 2 | 3 |
| Interpreting costs | 1 | 1 |
| What the core costs were based on | 1 | 1 |

Codes\\10. What policymakers want from the ENHANCE evaluation

| Name | Files | References |
| --- | --- | --- |
| Approval of government bodies | 1 | 1 |
| Describing the CEW population and the level of complexity | 1 | 1 |
| Documenting differences in service models | 1 | 1 |
| Economic analyses | 2 | 2 |
| Effectiveness or quality of care versus number of patients seen | 3 | 3 |
| Factors influencing numbers of patients seen | 1 | 2 |
| Hopes for the future | 0 | 0 |
| Clear pathway for obesity in every ICB | 1 | 1 |
| Guidance for local systems on decision making | 2 | 2 |
| Reducing weight stigma for young people | 1 | 1 |
| Scale up CEW, wider investment | 1 | 1 |
| Identifying the key components for a CEW clinic, including at different timepoints or ages | 3 | 3 |
| Inform future commissioning | 2 | 3 |
| Understanding effectiveness for different age groups | 1 | 1 |
| Understanding impact for young people, e.g. PROs or qual data | 1 | 1 |
| Understanding impact on complications | 2 | 2 |
| Understanding the wider social impacts, e.g. school attendance, labour market | 3 | 4 |
| Wider learning for the health service | 0 | 0 |
| How to build a service with a holistic approach around the patient | 1 | 1 |
| How to join up services, be more efficient | 1 | 1 |

Codes\\11. Thoughts on potential ways to group clinics for the analysis

| Name | Files | References |
| --- | --- | --- |
| MDT composition or interventions included in services | 1 | 1 |
| Tertiary (specialist) centre versus district general hospital | 2 | 3 |
