# Supplementary material for: From policy to person-centred care: insights into the development of complications of excess weight clinics in England
Source: BMC Health Serv Res. 2026 May 13;26:849. doi: 10.1186/s12913-026-14689-7 (PMC13281504; doi:10.1186/s12913-026-14689-7)
Supplement: Supplementary file 5 — Supplementary Material 5 [file 12913_2026_14689_MOESM5_ESM.docx]

ENHANCE policy interviews

Codes

Codes\\1. Involvement in CEW

| Name | Files | References |
| --- | --- | --- |
| Clinical lead for NHSE programme | 1 | 1 |
| Commissioned by NHSE to lead involvement work | 1 | 1 |
| Delphi | 2 | 2 |
| Establishing key goals of CEW | 1 | 2 |
| Importance of social and emotional outcomes to patients | 1 | 1 |
| Deputy Director for NHSE CYP Transformation Programme | 1 | 1 |
| Involve in bidding process for sites to be a part of CEW programme | 1 | 1 |
| Involved in education strategy group | 1 | 1 |
| Involved in funding allocations for clinics | 1 | 1 |
| Involved in scoping the need for CEW and getting the funding | 1 | 4 |
| Led on establishing the CEW programme | 1 | 1 |
| Part of NHSE CYP transformation team who originally set up CEW programme | 1 | 3 |
| Set up a childhood obesity clinic pre-CEW which fed into CEW | 2 | 2 |
| Undertook a gap analysis which highlighted no real guidance or consistency on chilhood obesity clinics | 1 | 1 |

Codes\\2. Reasons for developing national CEW programme (Inc aims and goals)

| Name | Files | References |
| --- | --- | --- |
| Aims and goals | 0 | 0 |
| Commitment to long term plan | 2 | 2 |
| Education strategy | 2 | 2 |
| Goals have been the same throughout | 1 | 1 |
| Improve the evidence base | 1 | 1 |
| Improving mental health and wellbeing | 2 | 2 |
| 'More than just weight' supporting young people with complications | 1 | 1 |
| Reducing weight stigma | 1 | 1 |
| Support quality of life not just clinical outcomes | 1 | 1 |
| Targets for numbers seen | 3 | 6 |
| Weight management (as primary focus) | 1 | 1 |
| Reasoning | 0 | 0 |
| Alleviate long-term strain on the health care system | 0 | 0 |
| Developing a service for a vulnerable cohort that have unmet need | 1 | 1 |
| Help raise awareness and galvanise local areas to do something about obesity | 1 | 1 |
| Prevention and Tier 2 have failed | 3 | 3 |
| Provide holistic and individualised care | 0 | 0 |
| Provide longer term support for young people | 0 | 0 |
| Test different approaches (physical and psychosocial care) | 0 | 0 |
| Test long term effectiveness of weight management | 1 | 1 |

Codes\\3. Delivery models (expected versus actual)

| Name | Files | References |
| --- | --- | --- |
| Actual delivery model | 0 | 0 |
| Already had a clinic in place so very little change | 1 | 1 |
| Anticipated national protocols and processes | 0 | 0 |
| Associated costs of delivery higher than anticipated (e.g. prescribing costs) | 0 | 0 |
| Currently exceeding expectations | 0 | 0 |
| Different age cut-offs, e.g. 16-18. | 1 | 2 |
| Implementation slower than expected | 1 | 1 |
| Merging of models | 1 | 1 |
| Multidisciplinary clinic that has evolved over time | 1 | 1 |
| People with more co-morbidities over time | 1 | 1 |
| Some clinics are medical heavy with limited MDT interventions | 1 | 1 |
| Tertiary versus district general hospital | 1 | 2 |
| Variable experience for different families | 1 | 1 |
| Variation in how delivered but similar holistic principles | 1 | 1 |
| Variation in number of patients being seen | 1 | 2 |
| Expected delivery model | 0 | 0 |
| Anticipate variation between CEW delivery models | 1 | 3 |
| Digitally focused approaches | 1 | 1 |
| Examples of workforce but not being too prescriptive | 2 | 2 |
| High level specification - leave the details up to sites | 2 | 3 |
| Better sustainability and local pride if developed locally | 1 | 1 |
| In order to meet targets, sites need to set up the way that works for them | 1 | 1 |
| It's a pilot | 1 | 1 |
| Limiting factor on variation is the finite funding | 1 | 1 |
| No evidence base to provide very specific guidance on how to set up | 2 | 3 |
| People couldn't agree on details but core principles agreed on | 1 | 1 |
| Holistic, biopsychosocial approach | 3 | 4 |
| Hub and spoke approach | 3 | 3 |
| Person and family centred care | 0 | 0 |
| Sharing learning within and between CEW clinics (Networking approaches) | 1 | 4 |
| Test alternative MDT approaches | 0 | 0 |

Codes\\4. Barriers to CEW development

| Name | Files | References |
| --- | --- | --- |
| Changing strategic oversight | 0 | 0 |
| Complexity of the CEW population | 0 | 0 |
| Clustering behaviours at family level | 0 | 0 |
| How safeguarding is handled with families is really important for engagement in the service | 1 | 2 |
| More complex than anticipated | 1 | 1 |
| COVID-19 pandemic | 1 | 1 |
| Creating a good quality data collection system | 1 | 1 |
| High demand for CEW programme, limited funding | 1 | 1 |
| Difficult to establish referral criteria due to so much need | 1 | 1 |
| Only so much you can do with limited funds | 1 | 1 |
| Lack of evidence | 1 | 1 |
| CEW data not easily accessible for local teams | 0 | 0 |
| How best to deliver a Tier 3 model | 1 | 2 |
| Lack of protocols for co-morbidity management | 0 | 0 |
| No previous clinic, had to start from scratch | 1 | 1 |
| Not suitable for specialised commissioning so needed time to set up | 1 | 1 |
| Postcode lottery with being able to prescribe GLP-1s | 1 | 2 |
| Shortage of workforce nationally | 2 | 4 |
| Short-term contracts (at clinic level and staff level) | 1 | 1 |
| Consistency of contracting | 1 | 1 |
| Inability to long term plan (ie. sustainability) | 2 | 3 |
| Loss of staff or staff turnover | 3 | 5 |
| Makes the services less resilient | 1 | 1 |
| The wider weight management system | 0 | 0 |
| Fragmented wraparound service provision (e.g. Tier 2) | 0 | 0 |
| Lack of historic funding in WM | 0 | 0 |
| Lack of WM infrastructure and pathways | 2 | 2 |
| Prevention narrative | 1 | 1 |
| Short-term thinking | 0 | 0 |
| The need to move to local commissioning | 1 | 1 |
| Time for regional CEW leads to be appointed | 1 | 1 |
| Unrealistic expectations of CEW (not perceived as a pilot programme by all) | 1 | 1 |

Codes\\5. Facilitators to CEW development

| Name | Files | References |
| --- | --- | --- |
| Autonomy of service development (Clinics able to shape their own service offer, ie. locally led) | 0 | 0 |
| Data and evidence illustrating need for CEW programme | 2 | 2 |
| Developing an education strategy alongside as was a new pathway | 1 | 1 |
| Easier if a clinic already existed before CEW | 1 | 3 |
| Importance of professional networks and sharing of lessons learnt to improve as we go | 1 | 2 |
| Multi-year funding commitment to CEW programme | 0 | 0 |
| National policy commitment to support CYP living with severe obesity | 0 | 0 |
| NHS Long Term Plan | 2 | 2 |
| There are interested people - long waiting lists | 2 | 2 |

Codes\\6. Alignment with wider policy context

| Name | Files | References |
| --- | --- | --- |
| Acknowledgement of previous failing of government childhood obesity initiatives | 1 | 1 |
| Changing contexts and governments | 1 | 1 |
| Decision making anticipated to lie with ICBs | 2 | 2 |
| Labour's child health action plan | 1 | 1 |
| Lack of national leadership | 0 | 0 |
| NHS Long Term Plan (&10 Year Health Plan) | 4 | 5 |
| Prevention narrative of national government | 1 | 3 |
| Wider obesity strategies, school interventions and sugar tax | 1 | 1 |

Codes\\7. Evaluation expectations

| Name | Files | References |
| --- | --- | --- |
| Clinical effectiveness of CEW services | 2 | 5 |
| Cost-effectiveness and -savings of CEW services | 3 | 9 |
| Desire to capture wider outcomes (ie. more than just BMI) | 3 | 4 |
| Determine complexity of CEW population | 2 | 2 |
| Evaluate different models of care (and core components - 'What works') | 4 | 6 |
| Evaluate equity of access | 1 | 2 |
| Findings to inform policy & decision making | 4 | 9 |
| How to join up services | 1 | 1 |
| Importance of flagging the importance of evidence generation | 1 | 1 |
| Importance of good data collection | 1 | 1 |
| Patient experience | 2 | 5 |
| Quality versus quantity of patients seen | 1 | 3 |
| Retention of patients within the CEW clinics | 1 | 1 |
| What works best for different age groups | 1 | 2 |
| Wider learning on how to build a service around the patient and take a holistic approach | 1 | 1 |

Codes\\8. Public role in shaping the CEW programme

| Name | Files | References |
| --- | --- | --- |
| Benefits of involving CYP and families | 0 | 0 |
| Confidence of CYP in presenting | 1 | 1 |
| CYP feeling listened to | 1 | 1 |
| Youth advisors created useful tools to inform practice | 1 | 1 |
| Have done qualitative work with patients instead of PPIE | 1 | 1 |
| Impact of involvement or PPIE work | 0 | 0 |
| Impacting the conversation about and awareness of mental health but possibly limited impact on what clinics did | 1 | 1 |
| Increased recognition of the importance of PPIE work but still some cultural change needed | 1 | 1 |
| PPIE lead helped shaped how questions were asked to CYP in the Delphi | 1 | 1 |
| Really highlighting the complexity of the patient group | 1 | 1 |
| Training session for CEW staff on how to do involvement work with the CEW population | 2 | 2 |
| Value of PPIE work not the same across all clinics | 1 | 1 |
| PPIE was also part of wider CYP transformation programme | 1 | 1 |
| Role of the involvement or PPIE work in CEW | 0 | 0 |
| Ongoing to underpin the whole programme | 1 | 1 |
| To ensure the voices of CYP and families brought into CEW development | 2 | 3 |
| Lack of confidence in having conversations with CYP with severe obesity | 1 | 2 |
| To make sure the clinics work | 1 | 1 |
| Stages and processes for involvement or PPIE work | 0 | 0 |
| 1st stage - Delphi | 4 | 5 |
| Clinical perspective - importance of including weight | 1 | 1 |
| Importance of mental health highlighted by CYP | 1 | 1 |
| 2nd stage - PPIE with the clinics | 2 | 4 |
| To understand what CYP and families think of the different CEW models | 2 | 2 |
| Importance of being able to build relationships with other CYP and families | 1 | 2 |
| Importance of youth and family workers | 1 | 2 |
| Title of professional less important than the skills of the professional | 1 | 1 |
| Type of model doesn't matter, the day-to-day details are important | 1 | 1 |
| 3rd stage - experience longer term | 1 | 1 |
| Lead working closely with NHSE and local areas | 1 | 1 |
| Online versus F2F engagement work | 1 | 1 |
| PPIE around the Evira technology | 1 | 1 |
| Recruitment to PPIE group | 1 | 1 |
| The need for skilled facilitator to do participation work with this cohort | 2 | 2 |
| Youth advisors | 1 | 1 |

Codes\\9. Hopes for the future of CEW

| Name | Files | References |
| --- | --- | --- |
| Build in engagement with CYP and families as a priority | 1 | 1 |
| Developing the service | 1 | 2 |
| Linking up with other youth services in the hospital | 1 | 1 |
| Peer-to-peer work | 1 | 1 |
| Encourage investment to scale up CEW services | 1 | 1 |
| Guidance for local systems to help them prioritise and design CEW work | 2 | 2 |
| Help de-stigmatise some of the lived experience and demonstrate the complexity | 1 | 1 |
| Integrated more seamlessly in the obesity pathway | 1 | 2 |
| Linking in with schools and other health providers to raise awareness of excess weight | 1 | 1 |
| Longer-term follow-up | 1 | 1 |
| Mental health at the forefront of teams | 1 | 1 |
| Original 21 services act as a focus for research and innovation | 1 | 1 |
| Prioritisation of youth worker or family support worker roles | 1 | 1 |
| Removing of silos - working more closely with other services involved with child | 1 | 1 |
| To have a clear pathway for obesity in every ICB | 1 | 1 |
| Transitioning to adult clinics | 1 | 1 |
